# Transcatheter arterial embolization enabling curative surgery for bleeding locally advanced breast cancer: a case report

**DOI:** 10.1097/RC9.0000000000000922

**Published:** 2026-09-08

**Authors:** Tomoe Kameyama, Tomoko Seki, Takamichi Yokoe, Aiko Nagayama, Maiko Takahashi, Tetsu Hayashida

**Affiliations:** Department of Breast Surgery, Keio University School of Medicine, Tokyo, Japan

**Keywords:** advanced breast cancer, angiography, endovascular therapy, hemorrhage, therapeutic embolization

## Abstract

**Introduction and importance:**

Locally advanced breast cancer (LABC), defined as stage IIIB or IIIC disease without distant metastasis, is generally managed with systemic therapy as the initial treatment. However, uncontrolled bleeding from fungating tumors may interrupt treatment continuity and render definitive surgery unfeasible. Although endovascular therapy is occasionally used as an emergency hemostatic measure, its role in enabling curative surgery remains insufficiently documented. We report a case of LABC in which transcatheter arterial embolization (TAE) successfully controlled tumor bleeding and enabled subsequent definitive resection.

**Presentation of case:**

A 66-year-old woman with untreated breast cancer presented with massive hemorrhage from a rapidly enlarging fungating tumor, resulting in transient loss of consciousness. Contrast-enhanced computed tomography revealed a 10-cm left breast mass with axillary and supraclavicular lymphadenopathy without distant metastasis (T4bN3cM0, stage IIIC). Persistent bleeding initially precluded definitive oncologic treatment. Endovascular embolization of the internal mammary and lateral thoracic arteries was performed on the second hospital day, achieving immediate and sustained hemostasis.

**Clinical discussion:**

Hemorrhage control enabled uninterrupted radiotherapy and endocrine therapy, leading to marked tumor regression. Subsequent total mastectomy with axillary dissection was performed without skin grafting, and pathology demonstrated minimal residual disease with no nodal metastasis.

**Conclusion:**

TAE may provide rapid and durable hemorrhage control, thereby allowing uninterrupted multimodal therapy and ultimately enabling curative surgery in selected patients with LABC.

## Introduction

Locally advanced breast cancer (LABC) is defined as stage IIIB or IIIC disease without distant metastasis and is characterized by extensive primary tumor invasion and/or regional lymph node involvement. Systemic therapy is the standard initial treatment, followed by surgery when feasible^[^1,2^]^. However, maintaining uninterrupted systemic therapy can be challenging, particularly in patients with uncontrolled tumor bleeding or excessive exudate from fungating lesions.

HIGHLIGHTS

- Transcatheter arterial embolization achieved rapid hemostasis in locally advanced breast cancer.
- Embolization enabled uninterrupted radiotherapy and endocrine therapy.
- Tumor regression allowed definitive surgery with curative-intent.
- Endovascular therapy may serve as a bridge from bleeding control to curative treatment.

Initial management includes compression or topical hemostatic agents, whereas severe or refractory bleeding may require endovascular therapy or surgery. Among these options, transcatheter arterial embolization (TAE) is especially advantageous in patients with unstable clinical conditions because it can be performed under local anesthesia with minimal invasiveness.

Herein, we report a case of LABC in which TAE achieved effective hemostasis, thereby enabling subsequent systemic therapy and an ultimately curative surgical resection of the primary tumor.

This case has been reported in accordance with the SCARE 2025 criteria^[^3^]^.

## Case report

### Clinical findings and diagnostic assessment

A 66-year-old woman with no significant medical history presented with progressive enlargement of a mass in the left breast. She had first noticed the mass approximately 10 years earlier but had not sought medical evaluation. Over the preceding 2 months, the tumor rapidly increased in size, resulting in two ulcerated lesions with malodorous exudate. On April 25 of Year X, sudden bleeding occurred from an ulcerated lesion, accompanied by transient loss of consciousness, prompting emergency transport to our hospital.

On admission, she was hemodynamically stable. Physical examination revealed a large, elevated 14 × 10-cm mass with a 3-cm ulcerated area on the left breast (Fig. 1A). Laboratory tests demonstrated anemia (hemoglobin 9.8 g/dL) without evidence of coagulopathy. However, due to repeated syncopal episodes associated with ongoing hemorrhage, four units of packed red blood cells were transfused.

**Figure 1.**
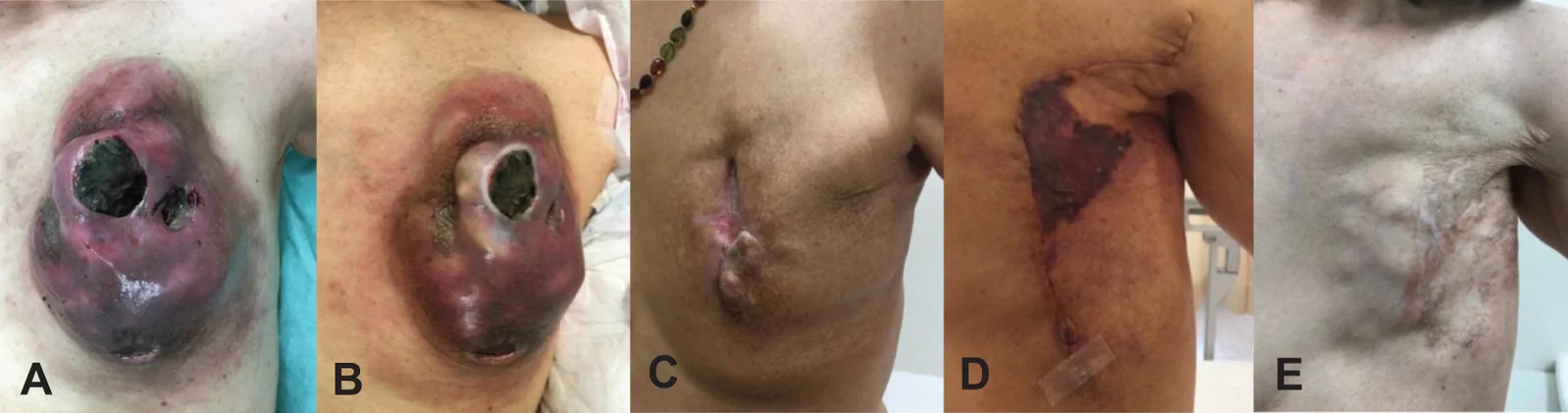
Local conditions. (A) At the initial visit; (B) Post-embolization; (C) Post-medication; (D) Post-operation; and (E) Seven years after the operation.

Brain computed tomography (CT) of the brain revealed no abnormalities. Contrast-enhanced CT of the chest demonstrated a 10-cm irregular breast mass with cutaneous ulceration, accompanied by enlarged left axillary and supraclavicular lymph nodes, without evidence of distant metastasis (cT4bN3cM0, cStage IIIC). Prominent feeding vessels arising from the left superior thoracic artery, internal mammary artery, and branches of the brachial artery were identified (Fig. 2). Given persistent bleeding and the risk of hemodynamic instability, urgent TAE was scheduled for the following day.

**Figure 2.**
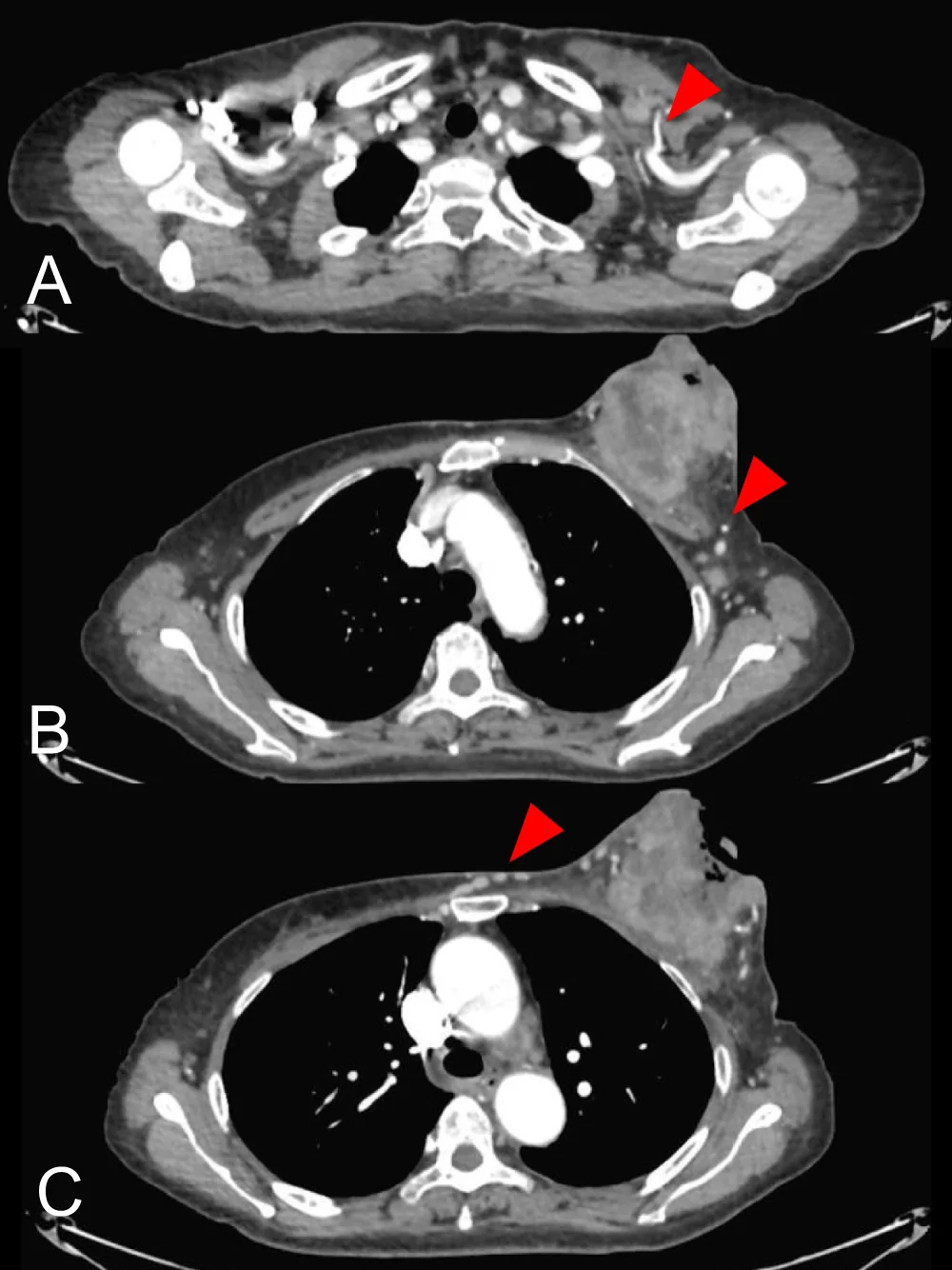
Axial contrast-enhanced computed tomography image obtained upon arrival. (A) Left superior thoracic artery; (B) Left brachial artery; and (C) Left internal mammary artery.

### Angiography and embolization procedure

Percutaneous access was obtained via the left brachial artery, and digital subtraction angiography was performed after the catheter was advanced to the left subclavian artery. The internal mammary artery and the lateral thoracic artery were identified as the primary tumor-feeding vessels. Two branches of the internal mammary artery and the lateral thoracic artery were selectively embolized using gelatin sponge particles (Serescue; Fig. 3). The procedure was completed without complications.

**Figure 3.**
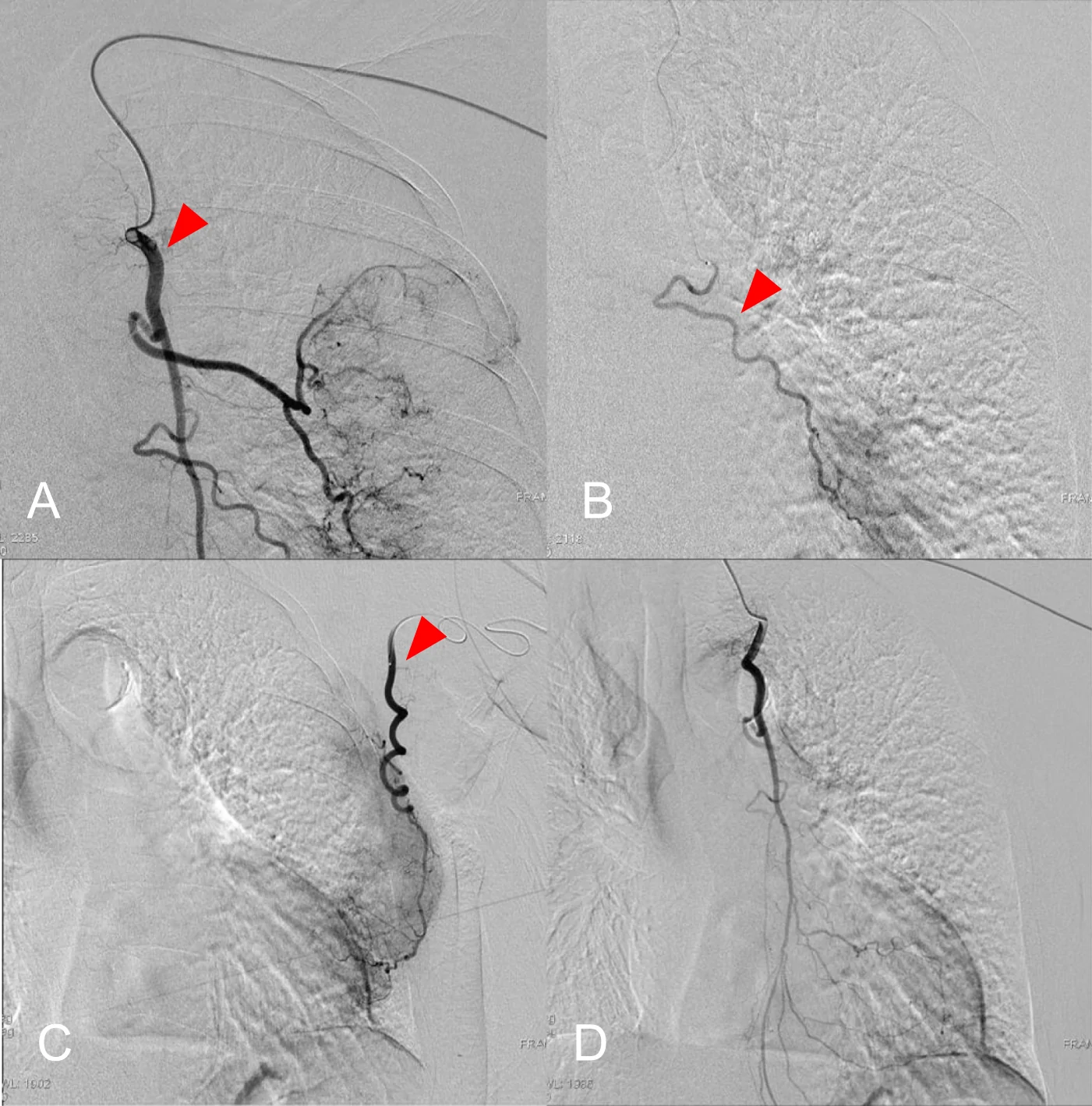
Angiographic findings: (A) Internal mammary artery; (B) Distal end of the internal mammary artery; (C) Lateral thoracic artery; and (D) After internal mammary artery embolization.

### Post-embolization course

Approximately 30 minutes after embolization, the ulcerated tumor surface gradually turned black, consistent with ischemic necrosis (Fig. 1B). The patient developed significant local pain, which was managed with analgesic therapy.

On May 2, radiotherapy was initiated for local control. Because the patient had no evidence of distant metastasis and remained a candidate for curative treatment, a total dose of 60 Gy in 25 fractions was prescribed to the breast to maximize durable local control. The supraclavicular region received the standard dose of 50 Gy in 25 fractions for regional nodal irradiation. Histopathological examination of a biopsy specimen obtained at admission confirmed invasive ductal carcinoma, nuclear grade I, estrogen receptor-positive (Allred score 5 + 3), progesterone receptor-positive (Allred score 4 + 2), HER2 score 1 +, and a Ki-67 index of 20%. Endocrine therapy with letrozole was initiated on May 9.

Both the primary tumor and the regional lymph node metastases gradually decreased in size. After approximately 12 months of neoadjuvant therapy, follow-up CT performed in March (Year X + 1) demonstrated that the primary tumor had decreased in size to approximately 5 cm (Fig. 1C), and definitive surgical resection was subsequently performed in May (Year X + 1). Before surgery, the patient was counseled that preoperative radiotherapy might increase operative difficulty because of treatment-related fibrosis, with potential risks of impaired wound healing and postoperative functional deficits.

### Surgical findings

On May 11 (Year X + 1), a total mastectomy with axillary lymph node dissection was performed. The skin was excised in an elliptical fashion encompassing the tumor, with an additional 3-cm skin incision toward the axilla to initiate the procedure. A circumferential full-thickness skin flap was created. Marked edema and neovascularization beneath the tumor required meticulous ligation.

During the axillary dissection, dense scar tissue was observed. The thoracodorsal artery, vein, and nerve were preserved, whereas the intercostobrachial nerve and long thoracic nerve could not be identified. The surgical wound was closed successfully without the need for a skin graft (Fig. 1D).

### Pathological findings and outcomes

Histological examination revealed only a small residual 1.2-mm invasive tumor, corresponding to a therapeutic response grade of 2b according to the Japanese Breast Cancer Society criteria. No residual lymph node metastasis was identified.

After surgery, letrozole was switched to tamoxifen because of arthralgia. Tamoxifen was subsequently discontinued because of suspected interstitial pneumonitis that required corticosteroid therapy. Although a concomitant herbal medicine was later considered the most likely causative agent, a causal relationship with tamoxifen could not be completely excluded. Exemestane was subsequently initiated but was also discontinued because of severe arthralgia. Given the patient’s intolerance to multiple endocrine agents and the previous episode of interstitial pneumonitis requiring corticosteroid therapy, the multidisciplinary team decided to withhold further endocrine therapy and continue careful surveillance (Supplemental Digital Content Table S1, available at: https://links.lww.com/IJSCR/A140).

Despite the inability to identify either the intercostobrachial nerve or the long thoracic nerve intraoperatively, no postoperative winged scapula, clinically significant sensory disturbance, or upper-extremity dysfunction was observed during follow-up.

The patient has undergone regular surveillance with tumor markers every 3 months and annual CT scans and bone scintigraphy. At the most recent follow-up, 7 years after the initial presentation, the patient remained clinically well, with no evidence of disease recurrence (Fig. 1E).

## Discussion

Management of bleeding in LABC is challenging. When conservative measures fail, TAE provides a minimally invasive option for rapid hemorrhage control, particularly in patients with a compromised general condition or initially unresectable disease. In the present case, the patient experienced transient loss of consciousness associated with hemorrhage from a rapidly enlarging fungating tumor. Although temporary hemostasis was achieved on admission, recurrent bleeding posed a potentially life-threatening risk and precluded the initiation of definitive oncologic treatment. Because no distant metastasis was identified and curative treatment remained feasible, TAE was selected to achieve definitive hemorrhage control rather than purely palliative management. Urgent mastectomy was considered to carry a substantial risk of excessive intraoperative bleeding because of the highly vascular tumor, while radiotherapy alone was unlikely to achieve sufficiently rapid hemostasis. Our strategy was to achieve prompt hemorrhage control, administer multimodal therapy, and perform surgery after maximal tumor regression whenever feasible. TAE, therefore, best supported this treatment strategy. Because the patient’s recent massive hemorrhage and transient hemodynamic instability increased the anticipated risk of cytotoxic chemotherapy, chemotherapy was not considered the optimal initial treatment. After successful hemostasis, radiotherapy was administered with curative intent to achieve durable local control before surgery. Favorable tumor biology further supported the use of endocrine therapy once the patient’s condition had stabilized.

The breast is supplied primarily by the internal mammary artery, lateral thoracic artery, anterior intercostal arteries, and thoracoacromial artery^[^4^]^. Consistent with these anatomical findings, the internal mammary artery and lateral thoracic artery were identified as the major tumor-feeding vessels in our patient.

A PubMed search using the terms “breast,” “bleeding,” and “embolization” identified eight previously reported cases^[^5–11^]^ (Table 1). Hemostasis was successfully achieved in all reported cases, demonstrating that embolization is an effective technique for controlling tumor-related hemorrhage. Most previously reported cases involved patients with recurrent or metastatic disease or those who were not considered suitable candidates for curative surgery. Consequently, embolization was used primarily as a palliative hemostatic intervention or to facilitate subsequent systemic therapy rather than as definitive surgical treatment. Although Atzori *et al*^[^9^]^ also reported curative surgery after embolization, their patient required repeated preoperative embolization, achieved only a modest response to neoadjuvant chemotherapy, and ultimately required skin graft reconstruction.

**Table 1:** Summary of reports on endovascular embolization for hemorrhage from breast cancer.

Year	Author	Author’s Department	Position	Primary/recurrent	Pathology	Hormone receptor	HER2	Clinical indication for embolization	Tumor feeder vessel	Embolic material	Complication	Subsequent curative surgery
1978^[^5**^]^**	D P Harrington	Radiology	Case 1 Left parasternal area	Recurrent	–	–	–	Life-threatening hemorrhage	Internal mammary artery	Autologous clot	–	No
			Case 2 Left chest wall	Recurrent	–	–	–	Persistent hemorrhage	Internal mammary artery	Oxycel + autologous clot + Ivalon	–	No
			Case 3 Right chest wall	Recurrent	Undifferentiated adenocarcinoma	–	–	Persistent hemorrhage	Thoracoacromial artery, lateral thoracic artery	Gelfoam	–	No
1988	E M Rankin	Surgery	–	–	–	–	–	–	–	–	–	**–**
2012^[^6^]^	John M Moriarty	Radiology	Left breast	Primary	Invasive ductal carcinoma	–	–	Life-threatening hemorrhage; unsuitable for surgery	Internal mammary artery	Embosphere	–	No
2016^[^7^]^	Şefika Aksoy	General surgery	Left axillary region	Recurrent	–	Negative	Negative	Life-threatening hemorrhage	Costocervical branch, internal mammary artery	Embosphere	**–**	No
2020^[^8^]^	Kishu Kitayama	Surgery	Right breast	Primary, Stage IV	–	Negative	Negative	Persistent hemorrhage	Lateral thoracic artery	Coil (embolization + TACE)	–	No
2023^[^9^]^	Giulia Atzori	Breast surgery	Right breast	Primary	Invasive ductal carcinoma	Negative	Negative	Life-threatening hemorrhage	Internal mammary artery	Microspheres	–	Yes
2024^[^10^]^	Thomas Le Tat	Radiology	Left breast	Primary	Pleomorphic lobular carcinoma	Positive	Negative	Life-threatening hemorrhage	Internal mammary artery	Embosphere	Pain	No
2025^[^11^]^	Ji Hwan Kang	Radiology	Left breast	Primary, Stage IV	–	–	–	Life-threatening hemorrhage	Internal mammary artery, **S**ubclavian branches	PVA	Fever, pain	No

By contrast, in the present case, TAE achieved immediate and sustained hemostasis, thereby allowing uninterrupted radiotherapy and endocrine therapy. This treatment strategy resulted in marked tumor regression and ultimately enabled definitive surgical resection with minimal tissue loss and without the need for skin grafting. These findings suggest that, in selected patients without distant metastasis, TAE may function as a critical bridge from emergent hemorrhage control to curative-intent multimodal treatment.

Potential complications include pain, necrosis, infection, and vascular access–related events; however, these were manageable and did not compromise subsequent treatment. Careful patient selection and multidisciplinary collaboration remain essential.

## Conclusion

This case demonstrates that TAE can facilitate uninterrupted multimodal therapy and subsequent curative surgical resection in selected patients with non-metastatic LABC. Beyond its established role in emergency hemostasis, TAE may also serve as an important component of curative-intent multidisciplinary treatment. Further studies are warranted to better define its optimal indications.

## Data Availability

The data supporting the findings of this case report are available from the corresponding author upon reasonable request.
